# (*M*, *N*)-Soft Intersection BL-Algebras and Their Congruences

**DOI:** 10.1155/2014/461060

**Published:** 2014-04-01

**Authors:** Xueling Ma, Hee Sik Kim

**Affiliations:** ^1^Department of Mathematics, Hubei Minzu University, Enshi, Hubei 445000, China; ^2^Department of Mathematics, Research Institute for Natural Sciences, Hanyang University, Seoul 133-791, Republic of Korea

## Abstract

The purpose of this paper is to give a foundation for providing a new soft algebraic tool in considering many problems containing uncertainties. In order to provide these new soft algebraic structures, we discuss a new soft set-(*M, N*)-soft intersection set, which is a generalization of soft intersection sets. We introduce the concepts of (*M, N*)-SI filters of BL-algebras and establish some characterizations. Especially, (*M, N*)-soft congruences in BL-algebras are concerned.

## 1. Introduction

It is well known that certain information processing, especially inferences based on certain information, is based on classical two-valued logic. In making inference levels, it is natural and necessary to attempt to establish some rational logic system as the logical foundation for uncertain information processing. BL-algebra has been introduced by Hájek as the algebraic structures for his Basic Logic [1]. A well-known example of a BL-algebra is the interval [0,1] endowed with the structure induced by a continuous *t*-norm. In fact, the MV-algebras, Gödel algebras, and product algebras are the most known classes of BL-algebras. BL-algebras are further discussed by many researchers; see [2–12].

We note that the complexities of modeling uncertain data in economics, engineering, environmental science, sociology, information sciences, and many other fields cannot be successfully dealt with by classical methods. Based on this reason, Molodtsov [13] proposed a completely new approach for modeling vagueness and uncertainty, which is called soft set theory. We note that soft set theory emphasizes a balanced coverage of both theory and practice. Nowadays, it has promoted a breath of the discipline of information sciences, intelligent systems, expert and decision support systems, knowledge systems and decision making, and so on. For example, see [14–24]. In particular, Ça$\overset{˘}{\text{g}}$man et al., Sezgin et al., and Jun et al. applied soft intersection theory to groups [25], near-rings [26], and BL-algebras [27], respectively.

In this paper, we organize the recent paper as follows. In Section 2, we recall some concepts and results of BL-algebras and soft sets. In Section 3, we investigate some characterizations of (*M*, *N*)-SI filters of BL-algebras. In particular, some important properties of (*M*, *N*)-soft congruences of BL-algebras are discussed in Section 4.

## 2. Preliminaries

Recall that an algebra *L* = (*L*, ≤, ∧, ∨, ⊙, →, 0,1) is a BL-*algebra* [1] if it is a bounded lattice such that the following conditions are satisfied:

- (*L*, ⊙, 1) is a commutative monoid;
- ⊙ and → form an adjoin pair; that is, *z* ≤ *x* → *y* if and only if *x*⊙*z* ≤ *y* for all *x*, *y*, *z* ∈ *L*;
- *x*∧*y* = *x*⊙(*x* → *y*);
- (*x* → *y*)∨(*y* → *x*) = 1.
- In what follows, *L* is a BL-algebra unless otherwise specified.
- In any BL-algebra *L*, the following statements are true (see [1, 5, 6]):
- *x* ≤ *y*⇔*x* → *y* = 1;
- *x* → (*y* → *z*) = (*x*⊙*y*) → *z* = *y* → (*x* → *z*);
- *x*⊙*y* ≤ *x*∧*y*;
- *x* → *y* ≤ (*z* → *x*)→(*z* → *y*), *x* → *y* ≤ (*y* → *z*)→(*x* → *z*);
- *x* → *x*′ = *x*′′ → *x*;
- *x*∨*x*′ = 1⇒*x*∧*x*′ = 0;
- (*x* → *y*)⊙(*y* → *z*) ≤ *x* → *z*;
- *x* ≤ *y*⇒*x* → *z* ≥ *y* → *z*;
- *x* ≤ *y*⇒*z* → *x* ≤ *z* → *y*,

where *x*′ = *x* → 0.

A nonempty subset *A* of *L* is called a* filter* of *L* if it satisfies the following conditions:

(I1) 1 ∈ *A*,
(I2) ∀*x* ∈ *A*, ∀*y* ∈ *L*, *x* → *y* ∈ *A*⇒*y* ∈ *A*.
 It is easy to check that a nonempty subset *A* of *L* is a filter of *L* if and only if it satisfies
(I3) ∀*x*, *y* ∈ *L*, *x*⊙*y* ∈ *A*,
(I4) ∀*x* ∈ *A*, ∀*y* ∈ *L*, *x* ≤ *y*⇒*y* ∈ *A* (see [6]).

From now on, we let *L* be a BL-algebra, *U* an initial universe, *E* a set of parameters, and *P*(*U*) the power set of *U* and *A*, *B*, *C*⊆*E*.

**Definition 1 (see [13, 16])**

A soft set *f*_*A*_ over *U* is a set defined by *f*_*A*_ : *E* → *P*(*U*) such that *f*_*A*_(*x*) = *∅* if *x* ∉ *A*. Here *f*_*A*_ is also called an* approximate function*. A soft set over *U* can be represented by the set of ordered pairs *f*_*A*_ = {(*x*, *f*_*A*_(*x*)) | *x* ∈ *E*, *f*_*A*_(*x*) ∈ *P*(*U*)}. It is clear to see that a soft set is a parameterized family of subsets of *U*. Note that the set of all soft sets over *U* will be denoted by *S*(*U*).

**Definition 2 (see [16])**

Let *f*_*A*_, *f*_*B*_ ∈ *S*(*U*).

1. *f*_*A*_ is said to be a* soft subset* of *f*_*B*_ and denoted by $f_{A}\tilde{\subseteq }f_{B}$ if *f*_*A*_(*x*)⊆*f*_*B*_(*x*), for all *x* ∈ *E*. *f*_*A*_ and *f*_*B*_ are said to be* soft equal*, denoted by *f*_*A*_ = *f*_*B*_, if $f_{A}\tilde{\subseteq }f_{B}$ and $f_{A}\tilde{⊇}f_{B}$.
2. The union of *f*_*A*_ and *f*_*B*_, denoted by $f_{A}\tilde{\cup }f_{B}$, is defined as $f_{A}\tilde{\cup }f_{B}=f_{A\cup B}$, where *f*_*A*∪*B*_(*x*) = *f*_*A*_(*x*) ∪ *f*_*B*_(*x*), for all *x* ∈ *E*.
3. The intersection of *f*_*A*_ and *f*_*B*_, denoted by $f_{A}\tilde{\cap }f_{B}$, is defined as $f_{A}\tilde{\cap }f_{B}=f_{A\cap B}$, where *f*_*A*∩*B*_(*x*) = *f*_*A*_(*x*)∩*f*_*B*_(*x*), for all *x* ∈ *E*.

**Definition 3 (see [27])**

A soft set *f*_*L*_ over *U* is called an SI-* filter* of *L* over *U* if it satisfies

 (*S*_1_)  *f*_*L*_(*x*)⊆*f*_*L*_(1) for any *x* ∈ *L*,
 (*S*_2_)  *f*_*L*_(*x* → *y*)∩*f*_*L*_(*x*)⊆*f*_*L*_(*y*) for all *x*, *y* ∈ *L*.

## 3. (*M*, *N*)-SI Filters

In this section, we introduce the concept of (*M*, *N*)-SI filters in BL-algebras and investigate some characterizations. From now on, we let *∅*⊆*M* ⊂ *N*⊆*U*.

**Definition 4**

A soft set *f*_*L*_ over *U* is called an (*M*, *N*)-*soft intersection filter* (briefly, (*M*, *N*)-SI* filter*) of *L* over *U* if it satisfies

(SI_1_) *f*_*L*_(*x*)∩*N*⊆*f*_*L*_(1) ∪ *M* for all *x* ∈ *L*,
(SI_2_) *f*_*L*_(*x* → *y*)∩*f*_*L*_(*x*)∩*N*⊆*f*_*L*_(*y*) ∪ *M* for all *x*, *y* ∈ *L*.

**Remark 5**

If *f*_*L*_ is an (*M*, *N*)-SI filter of *L* over *U*, then *f*_*L*_ is an (*∅*, *U*)-SI filter of *L* over *U*. Hence every SI-filter of *L* is an (*M*, *N*)-SI filter of *L*, but the converse need not be true in general. See the following example.

**Example 6**

Assume that *U* = *S*_3_, the symmetric 3-group is the universal set, and let *L* = {0, *a*, *b*, 1}, where 0 < *a* < *b* < 1. We define *x*∧*y* : = min⁡{*x*, *y*}, *x*∨*y* : = max⁡{*x*, *y*} and ⊙ and → as follows:

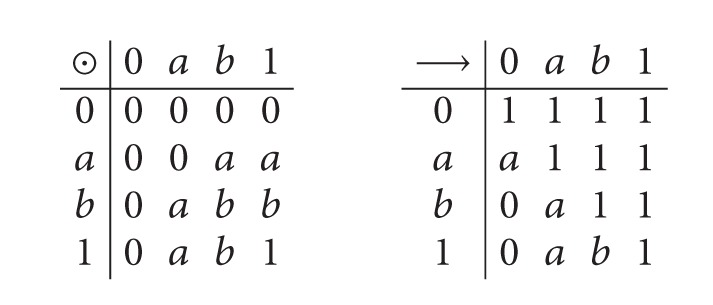
(1)
It is clear that (*L*, ∧, ∨, ⊙, →, 1) is a BL-algebra. Let *M* = {(13), (123)} and *N* = {(1), (12), (13), (123)}. Define a soft set *f*_*L*_ over *U* by *f*_*L*_(1) = {(1), (12), (123)}, *f*_*L*_(*b*) = {(1), (12), (13), (123)} and *f*_*L*_(*a*) = *f*_*L*_(0) = {(1), (12)}. Then we can easily check that *f*_*L*_ is an (*M*, *N*)-SI filter of *L* over *U*, but it is not SI-filter of *L* over *U* since *f*_*L*_(*b*)⊈*f*_*L*_(1).

The following proposition is obvious.

**Proposition 7**

If a soft set *f*_*L*_ over *U* is an (*M*, *N*)-*SI* filter of *L* over *U*, then

(2) $$\begin{array}{c} \left(f_{S}\left(1\right)\cap N\right)\cup M⊇\left(f_{S}\left(x\right)\cap N\right)\cup M\;\forall x\in S. \end{array}$$

Define an ordered relation “${\tilde{\subseteq }}_{\left(M,N\right)}$” on *S*(*U*) as follows: for any *f*_*L*_, *g*_*L*_ ∈ *S*(*U*), *∅*⊆*M* ⊂ *N*⊆*U*, we define $f_{L}{\tilde{\subseteq }}_{\left(M,N\right)}g_{L}\Leftrightarrow f_{L}\cap N\tilde{\subseteq }g_{L}\cup M$. And we define a relation “=_(*M*,*N*)_” as follows: $f_{L}{\;=\;}_{\left(M,N\right)}g_{L}\Leftrightarrow f_{L}{\tilde{\subseteq }}_{\left(M,N\right)}\;g_{L}$ and $g_{L}{\tilde{\subseteq }}_{\left(M,N\right)}\;f_{L}$. Using this notion we state Definition 4 as follows.

**Definition 8**

A soft set *f*_*L*_ over *U* is called an (*M*, *N*)-*soft intersection filter *(briefly, (*M*, *N*)-SI* filter*) of *L* over *U* if it satisfies

(SI_1_′) $f_{L}(x){\tilde{\subseteq }}_{\left(M,N\right)}f_{L}(1)$ for all *x* ∈ *L*,
(SI_2_′) $f_{L}(x\to y)\cap f_{L}(x){\tilde{\subseteq }}_{\left(M,N\right)}f_{L}(y)$ for all *x*, *y* ∈ *L*.

**Proposition 9**

If *f*_*L*_ is an (*M*, *N*)-SI filter of *L* over *U*, then *f*_L_* = {*x* ∈ *L* | (*f*_*L*_(*x*)∩*N*) ∪ *M* = (*f*_*L*_(1)∩*N*) ∪ *M*} is a filter of *L*.

**Proof**

Assume that *f*_*L*_ is an (*M*, *N*)-SI filter of *L* over *U*. Then it is clear that 1 ∈ *f*_L_*. For any *x*, *x* → *y* ∈ *f*_L_*, (*f*_*L*_(*x*)∩*N*) ∪ *M* = (*f*_*L*_(*x* → *y*)∩*N*) ∪ *M* = (*f*_*L*_(1)∩*N*) ∪ *M*. By Proposition 7, we have (*f*_*L*_(*y*)∩*N*) ∪ *M*⊆(*f*_*L*_(1)∩*N*) ∪ *M*. Since *f*_*L*_ is an (*M*, *N*)-SI filter of *L* over *U*, we have

(3) (fL(y)∩N)∪M=((fL(y)∪M)∩N)∪M⊇(fL(x)∩fL(x⟶y)∩N)∪M=((fL(y)∩N)∪M)∩((fL(x⟶y)∩N)∪M)=(fL(1)∩N)∪M.

Hence, (*f*_*L*_(*y*)∩*N*) ∪ *M* = (*f*_*L*_(1)∩*N*) ∪ *M*, which implies *y* ∈ *f*_L_*. This shows that *f*_L_* is a filter of *L*.

**Proposition 10**

If a soft set *f*_*L*_ over *U* is an (*M*, *N*)-*SI* filter of *L*, then for any *x*, *y*, *z* ∈ *L*,

1. $x\leq y\Rightarrow f_{L}(x){\tilde{\subseteq }}_{\left(M,N\right)}f_{L}(y)$,
2. $f_{L}(x\to y)=f_{L}(1)\Rightarrow f_{L}(x){\tilde{\subseteq }}_{\left(M,N\right)}f_{L}(y)$,
3. *f*_*L*_(*x*⊙*y*)=_(*M*,*N*)_ *f*_*L*_(*x*)∩*f*_*L*_(*y*)=_(*M*,*N*)_ *f*_*L*_(*x*∧*y*),
4. *f*_*L*_(0)=_(*M*,*N*)_ *f*_*L*_(*x*)∩*f*_*L*_(*x*′),
5. $f_{L}(x\to y)\cap f_{L}(y\to z){\tilde{\subseteq }}_{\left(M,N\right)}f_{L}(x\to z)$,
6. $f_{L}(x)\cap f_{L}(y){\tilde{\subseteq }}_{\left(M,N\right)}f_{L}(x⊙z\to y⊙z)$,
7. $f_{L}(x\to y){\tilde{\subseteq }}_{\left(M,N\right)}f_{L}((y\to z)\to (x\to z))$,
8. $f_{L}(x\to y){\tilde{\subseteq }}_{\left(M,N\right)}f_{L}((z\to x)\to (z\to y))$.

**Proof**

(1) Let *x*, *y* ∈ *L* be such that *x* ≤ *y*. Then *x* → *y* = 1, and hence

(4) (fL(x)∩N)=(fL(x)∩N)∩(fL(1)∪M)=(fL(y)∩N)∩(fL(x⟶y)∪M)⊆(fL(x)∩fL(x⟶y)∩N)∪M⊆fL(y)∪M,

which implies $f_{L}(x){\tilde{\subseteq }}_{\left(M,N\right)}f_{L}(y)$.

(2) Let *x*, *y* ∈ *L* be such that *f*_*L*_(*x* → *y*) = *f*_*L*_(1). Then,

(5) fL(x)∩N=(fL(x)∩N)∩(fL(1)∪M)=(fL(x)∩N)∩(fL(x⟶y)∪M)⊆(fL(x)∩fL(x⟶y)∩N)∪M⊆fL(y)∪M;

that is, $f_{L}(x){\tilde{\subseteq }}_{\left(M,N\right)}f_{L}(y)$.

(3) By (*a*_3_), we have *x*⊙*y* ≤ *x*∧*y* for all *x*, *y* ∈ *L*. By (1), $f_{L}(x⊙y){\tilde{\subseteq }}_{\left(M,N\right)}f_{L}(x)\cap f_{L}(y)$. Since *x* ≤ *y* → *x*⊙*y*, we obtain $f_{L}(x){\tilde{\subseteq }}_{\left(M,N\right)}f_{L}(y\to (x⊙y))$. It follows from (SI_2_) that $f_{L}(x)\cap f_{L}(y){\tilde{\subseteq }}_{\left(M,N\right)}f_{L}(y\to (x⊙y))\cap f_{L}(y)\subseteq f_{L}(x⊙y)$. Hence, *f*_*L*_(*x*⊙*y*)  =_(*M*,*N*)_ *f*_*L*_(*x*)∩*f*_*L*_(*y*).

Since *y* ≤ *x* → *y* and *x*⊙(*x* → *y*) ≤ *x*∧*y*, we have $f_{L}(y){\tilde{\subseteq }}_{\left(M,N\right)}f_{L}(x\to y)$ and $f_{L}(x⊙(x\to y)){\tilde{\subseteq }}_{\left(M,N\right)}f_{L}(x\wedge y)$. Hence we have

$f_{L}(x)\cap f_{L}(y){\tilde{\subseteq }}_{\left(M,N\right)}f_{L}(x)\cap f_{L}(x\to y){=}_{\left(M,N\right)}f_{L}(x⊙(x\to y)){\tilde{\subseteq }}_{\left(M,N\right)}f_{L}(x\wedge y){\tilde{\subseteq }}_{\left(M,N\right)}f_{L}(x)\cap f_{L}(y)$, which implies *f*_*L*_(*x*)∩*f*_*L*_(*y*)=_(*M*,*N*)_ *f*_*L*_(*x*∧*y*). Thus *f*_*L*_(*x*⊙*y*)=_(*M*,*N*)_ *f*_*L*_(*x*)∩*f*_*L*_(*y*)=_(*M*,*N*)_ *f*_*L*_(*x*∧*y*).

(4) It is a consequence of (3), since *x*⊙*x*′ = 0.

(5) By (*a*_4_).

(6) By (*a*_7_).

(7) By (*a*_8_).

(8) By (*a*_9_).

By Definition 4 and Proposition 10, we can deduce the following result.

**Proposition 11**

A soft set *f*_*L*_ over *U* is an (*M*, *N*)-*SI* filter of *L* over *U* if and only if it satisfies

(6) $$\begin{array}{c} \left(SI_{3}\right)x⟶\left(y⟶z\right)=1⟹f_{L}\left(x\right)\cap f_{L}\left(y\right){\tilde{\subseteq }}_{\left(M,N\right)}f_{L}\left(z\right). \end{array}$$

**Proposition 12**

A soft set *f*_*L*_ over *U* is an (*M*, *N*)-*SI* filter of *L* over *U* if and only if it satisfies

 
$\left(SI_{4})\;\forall x,y\in L,x\leq y\;\Rightarrow f_{L}(x){\tilde{\subseteq }}_{\left(M,N\right)}f_{L}(y\right)$,
 (*SI*_5_)  ∀ *x*, *y* ∈ *L*, *f*_*L*_(*x*⊙*y*)=_(*M*,*N*)_ *f*_*L*_(*x*)∩*f*_*L*_(*y*).

**Proof**

(⇒) By Proposition 10(1) and (3).

(⇐) Let *x*, *y* ∈ *L*. Since *x* ≤ 1, by (SI_3_), we have $f_{L}(x){\tilde{\subseteq }}_{\left(M,N\right)}f_{L}(1)$. Hence (SI_1_′) holds. Since *x*⊙(*x* → *y*) ≤ *y*, by (SI_3_) and (SI_4_), we have $f_{L}(x)\cap f_{L}(x\to y){=}_{\left(M,N\right)}f_{L}(x⊙(x\to y)){\tilde{\subseteq }}_{\left(M,N\right)}f_{L}(y)$; that is, (SI_2_′) holds. Therefore, *f*_*L*_ is an (*M*, *N*)-SI filter of *L* over *U*.

## 4. (*M*, *N*)-Soft Congruences

In this section, we investigate (*M*, *N*)-soft congruences, (*M*, *N*)-soft congruences classes, and quotient soft BL-algebras.

**Definition 13**

A soft relation *θ* from *f*_*L*_ × *f*_*L*_ to *P*(*U* × *U*) is called an (*M*, *N*)-*congruence* in *L* over *U* × *U* if it satisfies

 (*C*_1_)  *θ*(1,1)=_(*M*,*N*)_ *θ*(*x*, *x*), ∀*x* ∈ *L*,
 (*C*_2_)  *θ*(*x*, *y*)=_(*M*,*N*)_ *θ*(*y*, *x*), ∀*x* ∈ *L*,
 
$(C_{3})\;\theta (x,y)\cap \theta (y,z){\tilde{\subseteq }}_{\left(M,N\right)}\theta (x,z),\forall x,y,z\in L$,
 
$(C_{4})\;\theta (x,y){\tilde{\subseteq }}_{\left(M,N\right)}\theta (x⊙z,y⊙z),\forall x,y,z\in L$,
 
$(C_{5})\;\theta (x,y){\tilde{\subseteq }}_{\left(M,N\right)}\theta (x\to z,y\to z)\cap \theta (z\to x,z\to y),\forall x,y,z\in L$.

**Definition 14**

Let *θ* be an (*M*, *N*)-congruence in BL-algebra *L* over *U* × *U* and *x* ∈ *L*. Define *θ* ^*x*^ in *L* as *θ* ^*x*^(*y*) = *θ*(*x*, *y*), ∀*y* ∈ *L*. The set *θ* ^*x*^ is called an (*M*, *N*)-*congruence class* of *x* by *θ* in *L*. The set *L*/*θ* = {*θ* ^*x*^ | *x* ∈ *L*} is called a* quotient soft set* by *θ*.

**Lemma 15**

If *θ* is an (*M*, *N*)-congruence in *L* over *U* × *U*, then $\theta (x,y){\tilde{\subseteq }}_{\left(M,N\right)}\theta (1,1),\forall x,y\in L$.

**Proof**

By (*C*_1_) and (*C*_3_), we have $\theta (1,1)=\theta (x,x){\tilde{⊇}}_{\left(M,N\right)}\theta (x,y)\cap \theta (y,x)=\theta (x,y)$.

**Lemma 16**

If *θ* is an (*M*, *N*)-congruence in *L* over *U* × *U*, then *θ* ^1^ is an (*M*, *N*)-*SI* filter of *L* over *U*.

**Proof**

For any *x* ∈ *L*, we have

(7) $$\begin{array}{c} \theta^{1}\left(1\right)=\theta \left(1,1\right){\tilde{⊇}}_{\left(M,N\right)}\theta \left(1,x\right)=\theta^{1}\left(x\right). \end{array}$$

This proves that (SI_1_′) holds.

For any *x*, *y* ∈ *L*, by (*C*_3_) and (*C*_5_), we obtain

(8) $$\begin{array}{c} \theta \left(1,y\right){\tilde{⊇}}_{\left(M,N\right)}\theta \left(1,x⟶y\right)\cap \theta \left(x⟶y,y\right),\\ \theta \left(x⟶y,y\right)=\theta \left(x⟶y,1⟶y\right){\tilde{⊇}}_{\left(M,N\right)}\theta \left(x,1\right). \end{array}$$

It follows that

(9) θ(1,y)⊇~(M,N)θ(1,x⟶y)∩θ(x,1)=θ(1,x)∩θ(1,x⟶y);

that is, $\theta^{1}(y){\tilde{⊇}}_{\left(M,N\right)}\theta^{1}(x)\cap \theta^{1}(x\to y)$. This proves that (SI_2_′) holds. Thus, *θ* ^1^ is an (*M*, *N*)-SI filter of *L* over *U*.

**Lemma 17**

Let *f*_*L*_ be an (*M*, *N*)-SI filter of *L* over *U*. Then *θ*(*x*, *y*) = *f*_*L*_(*x* → *y*)∩*f*_*L*_(*y* → *x*) is an (*M*, *N*)-soft congruence in *L*.

**Proof**

For any *x*, *y*, *z* ∈ *L*, we have the following.

 (*C*_1_) Consider
  (10) θf(1,1)=fL(1⟶1)∩fL(1⟶1)=fL(1)=fL(x⟶x)∩fL(x⟶x)=θf(x,x).

This proves that (*C*_1_) holds.

 (*C*_2_) It is clear that (*C*_2_) holds.
 (*C*_3_) By Proposition 10(5), we have
  (11) θf(x,y)∩θf(y,z)  =(fL(x⟶y)∩fL(y⟶x))  ∩(fL(y⟶z)∩fL(z⟶y))  =(fL(x⟶y)∩fL(y⟶z))  ∩(fL(y⟶x)∩fL(z⟶y))  ⊆~(M,N)fL(x⟶z)∩fL(z⟶x)  =θf(x,z).

Thus (*C*_3_) holds.

(*C*_4_) Since *x* → *y* ≤ (*x*⊙*z*)→(*y*⊙*z*) and *y* → *x* ≤ (*y*⊙*z*)→(*x*⊙*z*), we have

(12) $$\begin{array}{c} f_{L}\left(x⟶y\right){\tilde{\subseteq }}_{\left(M,N\right)}f_{L}\left(\left(x⊙z\right)⟶\left(y⊙z\right)\right),\\ f_{L}\left(y⟶z\right){\tilde{\subseteq }}_{\left(M,N\right)}f_{L}\left(\left(y⊙z\right)⟶\left(x⊙z\right)\right). \end{array}$$

Thus, we have

(13) fL(x⟶y)∩fL(y→x)⊆~(M,N)fL((x⊙z)⟶(y⊙z))∩fL((y⊙z)⟶(x⊙z)),

which implies

(14) $$\begin{array}{c} \theta_{f}\left(x,y\right){\tilde{\subseteq }}_{\left(M,N\right)}\theta_{f}\left(x⊙z,y⊙z\right). \end{array}$$

This implies that (*C*_4_) holds.

(*C*_5_) Finally, we prove condition (*C*_5_):

(15) θf(x⟶z,y⟶z)∩θf(z⟶x,z⟶y)  =fL((x⟶z)⟶(y⟶z))  ∩fL((y⟶z)⟶(x⟶z))  ∩fL((z⟶x)⟶(z⟶y))  ∩fL((z⟶y)⟶(z⟶x))  ⊇~(M,N)fL(y⟶x)∩fL(x⟶y)  =θf(x,y).

Thus, (*C*_5_) holds. Therefore *θ*_*f*_ is an (*M*, *N*)-soft congruence in *L*.

Let *f*_*L*_ be an (*M*, *N*)-SI filter of *L* over *U* and *x* ∈ *L*. In the following, let *f* ^*x*^ denote the (*M*, *N*)-congruence class of *x* by *θ*_*f*_ in *L* and let *L*/*f* be the quotient soft set by *θ*_*f*_.

**Lemma 18**

If *f*_*L*_ is an (*M*, *N*)-*SI* filter of *L* over *U*, then *f* ^*x*^=_(*M*,*N*)_ *f* ^*y*^ if and only if *f*_*L*_(*x* → *y*)=_(*M*,*N*)_ *f*_*L*_(*y* → *x*)=_(*M*,*N*)_ *f*_*L*_(1) for all *x*, *y* ∈ *L*.

**Proof**

If *f*_*L*_ is an (*M*, *N*)-SI filter of *L* over *U*, then *f* ^*μ*^(*ν*) = *θ*_*f*_^*μ*^(*ν*) = *θ*_*f*_(*μ*, *ν*) = *f*_*L*_(*μ* → *ν*)∩*f*_*L*_(*ν* → *μ*); that is, *f* ^*μ*^(*ν*) = *f*_*L*_(*μ* → *ν*)∩*f*_*L*_(*ν* → *μ*) for all *x*, *y* ∈ *L*. If *f* ^*x*^=_(*M*,*N*)_ *f* ^*y*^, then *f* ^*x*^(*x*)=_(*M*,*N*)_ *f* ^*y*^(*x*), and hence *f*_*L*_(*x* → *x*) = *f*_*L*_(1)=_(*M*,*N*)_ *f*_*L*_(*y* → *x*)∩*f*_*L*_(*x* → *y*). Thus, *f*_*L*_(*y* → *x*)=_(*M*,*N*)_ *f*_*L*_(*x* → *y*)=_(*M*,*N*)_ *f*_*L*_(1).

Conversely, assume the given condition holds. By Proposition 10, we have $f_{L}(x\to z){\tilde{⊇}}_{\left(M,N\right)}\;f_{L}(x\to y)\cap f_{L}(y\to z)$ and $f_{L}(y\to z){\tilde{⊇}}_{\left(M,N\right)}f_{L}(y\to x)\cap f_{L}(x\to z)$. If *f*_*L*_(*y* → *x*)=_(*M*,*N*)_ *f*_*L*_(*x* → *y*)=_(*M*,*N*)_ *f*_*L*_(1), then *f*_*L*_(*x* → *z*)⊇_(*M*,*N*)_ *f*_*L*_(*y* → *z*) and *f*_*L*_(*y* → *z*)⊇_(*M*,*N*)_ *f*_*L*_(*x* → *z*). Thus *f*_*L*_(*x* → *z*)=_(*M*,*N*)_ *f*_*L*_(*y* → *z*). Similarly, we can prove that *f*_*L*_(*z* → *x*)=_(*M*,*N*)_ *f*_*L*_(*z* → *y*). This implies that

(16) fx(z)=fL(x⟶z)∩fL(z⟶x)=(M,N)fL(y⟶z)∩fL(z⟶y)=fLy(z),

for all *z* ∈ *L*. Hence, *f* ^*x*^=_(*M*,*N*)_ *f* ^*y*^.

We denote *f*_*f*(1)_ by *f*_*f*(1)_ : = {*x* ∈ *L* | *f*(*x*)=_(*M*,*N*)_ *f*(1)}.

**Corollary 19**

If *f* is an (*M*, *N*)-*SI* filter of *L* over *U*, then *f* ^*x*^=_(*M*,*N*)_ *f* ^*y*^ if and only if *x*~_*f*_*f*(1)__ *y*, where *x*~_*f*_*f*(1)__ *y* if and only if *x* → *y* ∈ *f*_*f*(1)_ and *y* → *x* ∈ *f*_*f*(1)_.

Let *f* be an (*M*, *N*)-*SI* filter of *L* over *U*. For any *f* ^*x*^, *f* ^*y*^ ∈ *L*/*f*, we define

(17) fx∨fy=(M,N)fx∨y,  fx∧fy=(M,N)fx∧y,fx⊙fy=(M,N)fx⊙y,  fx⟶fy=(M,N)fx→y.

**Theorem 20**

If *f* is an (*M*, *N*)-*SI* filter of *L* over *U*, then *L*/*f* = (*L*/*f*, ∧, ∨,′, →, *f* ^0^, *f* ^1^) is a BL-algebra.

**Proof**

We claim that the above operations on *L*/*f* are well defined. In fact, if *f* ^*x*^=_(*M*,*N*)_ *f* ^*y*^ and *f* ^*a*^=_(*M*,*N*)_ *f* ^*b*^, by Corollary 19, we have *x*~_*f*_(*f*(1))__ *y* and *a*~_*f*_*f*(1)__ *b*, and so *x*∨*a*~_*f*_*f*(1)__ *y*∨*b*. Thus *f* ^*x*∨*a*^=_(*M*,*N*)_ *f* ^*y*∨*b*^. Similarly, we prove *f* ^*x*∧*a*^=_(*M*,*N*)_ *f* ^*y*∧*b*^, *f* ^*x*⊙*a*^=_(*M*,*N*)_ *f* ^*y*⊙*b*^, and *f* ^*x*→*a*^=_(*M*,*N*)_ *f* ^*y*→*b*^. Then it is easy to see that *L*/*f* is a BL-algebra. Especially, we prove the divisibility in *L*/*f* as follows. Define a lattice ordered relation “≼_(*M*,*N*)_” on *L*/*f* as follows:

(18) $$\begin{array}{c} f^{x}{≼}_{\left(M,N\right)}f^{y}⟺f^{x}\vee f^{y}{=}_{\left(M,N\right)}f^{1}. \end{array}$$

By Corollary 19, we have *f*_*L*_(*x* → *y*)=_(*M*,*N*)_ *f*_*L*_(1). If *f* ^*x*^, *f* ^*y*^, *f* ^*z*^ ∈ *L*/*f*, then

(19) fx⊙fy≼(M,N)fz⟺fx⊙y≼(M,N)fz⟺fL((x⊙y)⟶z)=(M,N)fL(1)⟺fL(x→(y⟶z))=(M,N)fL(1)⟺fx≼(M,N)fy→z⟺fx≼(M,N)fy⟶fz.

**Theorem 21**

If *f*_*L*_ is an (*M*, *N*)-*SI* filter of *L* over *U*, then *L*/*f*≅*L*/*f*_*f*(1)_.

**Proof**

Define *φ* : *L* → *L*/*f* by *φ*(*x*) = *f* ^*x*^ for all *x* ∈ *L*. For any *x*, *y* ∈ *L*, we have

(20) φ(x∨y)=fx∨y=(M,N)fx∨fy=φ(x)∨φ(y),φ(x∧y)=fx∧y=(M,N)fx∧fy=φ(x)∧φ(y),φ(x⊙y)=fx⊙y=(M,N)fx⊙fy=φ(x)⊙φ(y),φ(x⟶y)=fx→y=(M,N)fx⟶fy=φ(x)⟶φ(y).

Hence, *φ* is an epic. Moreover, we have

(21) x∈Ker⁡φ  ⟺φ(x)=f1⟺fx=(M,N)f1⟺x∼ff(1)1⟺x∈ff(1),

which shows that *L*/*f*≅*L*/*f*_*f*(1)_.

## 5. Conclusions

As a generalization of soft intersection filters of BL-algebras, we introduce the concept of (*M*, *N*)-SI (implicative) filters of BL-algebras. We investigate their characterizations. In particular, we describe (*M*, *N*)-soft congruences in BL-algebras.

To extend this work, one can further investigate (*M*, *N*)-SI prime (semiprime) filters of BL-algebras. Maybe one can apply this idea to decision making, data analysis, and knowledge based systems.
